# Novel in Vitro Efficiency of Chitosan Biomolecule against *Trichomonas gallinae*

**Published:** 2012

**Authors:** M Tavassoli, A Imani, H Tajik, M Moradi, SH Pourseyed

**Affiliations:** 1 Department of Pathobiology, Faculty of Veterinary Medicine, Urmia University, Urmia, Iran; 2 Department of Food Science and Technology, Ahar Faculty of Agriculture, University of Tabriz, Tabriz, Iran

**Keywords:** *Trichomonas gallinae*, Chitosan, Antiparasitic drugs, Trophozoite, In Vitro

## Abstract

**Background:**

Development of new natural agents for parasitic diseases treatment has unexpectedly increased to overcome effectively against emergence and re-emergence of parasitic diseases, the appearance of drug resistant organisms and toxic side effects of current agents. The aim of the study was to evaluate antiprotozoal activities of chitosan biomolecule on trophozoites of *Trichomonas gallinae*.

**Methods:**

The antitrichomonal activity of various low molecular weight chitosan concentrations including 125, 250, 500 and 1250 µg ml^−1^ against *T. gallinae* trophozoites cultured in trypticase-yeast extract-maltose medium supplemented with heat-inactivated cold horse serum was evaluated in vitro. Samples containing medium without chitosan were also assayed as controls.

**Results:**

The mortality rates at 0, 3 and 6 h post treatment with all concentrations were significantly different from control group (*P*<0.05). Treated trophozoites showed more susceptibility to the highest concentration reaching mortality rate of 100% at 3h post inoculation. However, at this time, results for 125, 250 and 500 µg ml^−1^ were 93%, 95% and 96.7%, respectively.

**Conclusion:**

The results demonstrate that the application of chitosan biomolecule is a promising option for treatment of trichomoniasis in pigeons.

## Introduction

In recent years, application of synthetic antiparasitic drugs has been criticized by many researchers and animal health care professionals because of some drawbacks including, the emergence and re-emergence of parasitic diseases, the appearance of drug resistant organisms and toxic side effects (1). As a result, the development of new natural agents for animal parasitic diseases treatment has unexpectedly increased to overcome these problems. Among them, plant and animal based naturally produced agents can be used as alternatives or adjuncts to current antiparasitic therapies (2, 3).

Avian trichomoniasis is caused by a single celled protozoan *T. gallinae* which is mainly responsible for infections of mouth pharynx, esophagus, and crop of domestic pigeons, wild columbids and raptons (4). It is likely that *T. gallinae* is found wherever domestic pigeons and mourning doves are found (5).

**Fig. 1:**
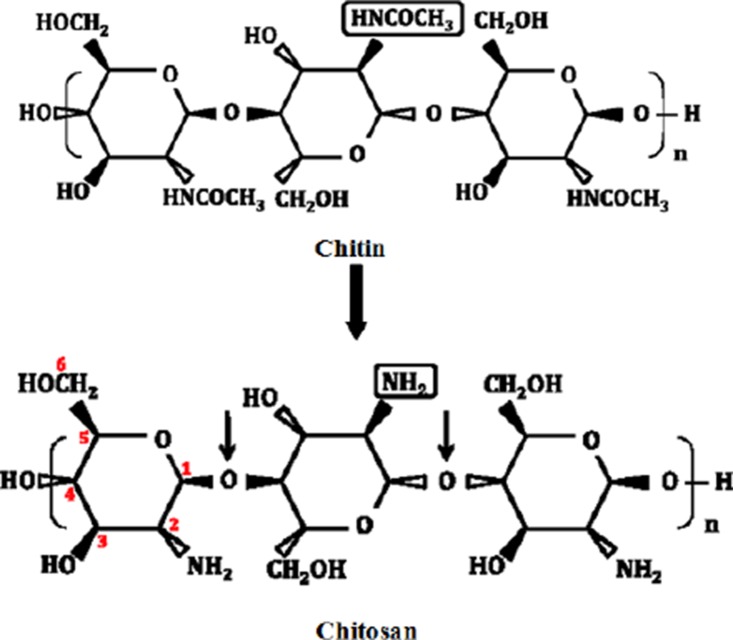
Structures of chitin and chitosan

Chitosan is a linear co-polymer of glucosamine (2-desoxy-2-amino-β-D-glucopyranose, glcN) and N-acetylglucosamine (2-desoxy-2-acetamido-β-D-glucopyranose, glcNAc) in β -1,4-glycosidic linkages (4). It is converted from chitin, which is a natural carbohydrate polymer found in the skeleton of crustaceans, marine zooplankton, insects and cell walls of fungi (6). Chitosan is found in nature, to a lesser extent than chitin, in the cell walls of fungi. In contrast to cellulose (the fiber-forming component of plant cell walls) and chitin (the fiber-forming component of fungal cell walls), chitosan possess positive charges at physiological pH values due to their free amino groups (Fig. 1) (7). Among abundant naturally occurring polysaccharides, which are neutral or acidic, chitosan is distinguished for its cationic nature (pK_a_ ≈ 6.5), which allows it to interact with the many polyanionic polymers such as proteins, DNA, and membranes (8). Chitosan possesses many desirable properties including, antimicrobial, antifungal and antioxidant activity and also film forming properties which have been reviewed in great details (9). Moreover, as reported earlier (10, 11), chitosan and its derivatives could consider as a novel antiparasitic agent.

The efficacy of several nitroimidazoles such as metronidazole, dimetridazole, ronidazole, carnidazole and ornidazole against *T. gallinae* has been reported (12). In addition, the susceptibility of this protozoan to four synthetic compounds was evaluated in vitro (13). The aim of the present study was to evaluate possible antiprotozoal activities of various concentrations of low molecular weight chitosan on trophozoites of *T. gallinae* in vitro.

## Materials and Methods

### Chitosan solution preparation

Chitosan solution was prepared by dissolving chitosan with low molecular weight (Sigma, Aldrich, St. Louis, MO, USA) in aqueous solution of 1% (w/v) acetic acid to a final concentration of 20000 µgml^−1^ with agitation overnight at room temperature to achieve complete chitosan dispersion. The pH of solution was then adjusted to 7 with 1 mol equi/L NaOH.

### Parasite collection and culture

In this study which conducted at Laboratory of Parasitology (Faculty of Veterinary Medicine, Urmia University, Urmia, Iran,), *T. gallinae* isolated from an adult pigeon was cultured axenically in vitro in trypticase-yeast extract-maltose (TYM) medium (pH 7.2) supplemented with 10% (v/v) heat-inactivated cold horse serum (14), and passaged every two weeks. Stock suspensions of 1 ×10^5^ trophozoites ml^−1^ in the caped vials were used.

### Dose response bioassay

Based on our preliminary experiment, from stock chitosan solution, different concentrations of chitosan including: 1250, 500, 250 and 125 µg ml^−1^ (equivalent to 0.125, 0.05, 0.025 and 0.0125% respectively) were prepared in stock suspensions of parasites. The control samples without chitosan were prepared as the same procedure discussed in chitosan solution preparation.

### Viability assessment

Vials of treated samples were kept at 37°C and observed at 0, 3 and 6h post treatment (PT) using a light microscope to check dead trophozoites by assessing their motility. Assessment was carried out in triplicate for each concentration as well the control and the percentage mortality was recorded. Samples containing medium without chitosan were also assayed as negative controls.

### Statistical analysis

All viability measurements were carried out in triplicate and the average of mortality rates were transformed to logarithms and submitted to analysis of variance using Generalised Linear Model and Repeated Measure Analysis (two-factor mixed model) using (SPSS 17.0 for Windows, SPSS Inc., Chicago, IL) for observation of significant variation among them. Values of *P* < 0.05 were considered significantly different.

## Results

**Fig. 2:**
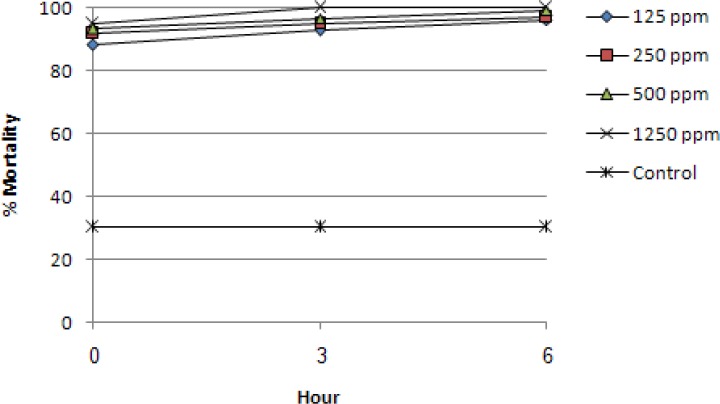
Effect of different concentrations of chitosan on the mortality rate of *T. gallinae* trophozoites. Control was samples containing medium without chitosan

We first investigated the possible effects of low molecular weight chitosan on the mortality/survival of *T. gallinae* trophozoites in vitro. The results for the toxicity of different concentrations (1250, 500, 250 and 125 µg ml^−1^) of chitosan at 0, 3 and 6h PT are shown in Fig. 2. The final solvent concentration, that is the highest concentration, was achieved by a preliminary experiment. The results of this work demonstrated that there were significant differences (*P* < 0.05) between the control samples and four different concentrations of chitosan at 0, 3 and 6h PT. As illustrated in Fig. 2, after 6h of exposure, all chitosan concentrations showed high mortality rate ranging from 96.3% to 100% which were significantly (*P* < 0.05) different form the control group (30%). Moreover, among chitosan used dosages, there was significant difference between concentrations at 0 and 6h, whereas, at 0 and 3h or 3 and 6h, it was not significant (*P* < 0.05). The maximum trophozoites mortality rate (100%) was observed at 1250 µg ml^−1^ concentration after 3h PT.

## Discussion

In this study, we preliminary demonstrated that low molecular weight chitosan was promising in creating new anti-protozoal effective agent in vitro against *T. gallinae*. Although the exact mechanism of antiprotozoal activity of chitosan is not fully elucidated, but nevertheless, ability to induce morphological changes in the cell parasite, in part, may be a possible mechanism. Only a few attempts have been made to determine antiparasitic properties of chitosan. In one of them (11) investigated the effects of chitosan microspheres cross linked with glutaraldehyde and containing beta-cyclodextrin (*β*CD) on the survival of *Philasterides dicentrarchi*, a protozoan ciliate, in 7d cultures. When used alone in assays, chitosan (10 µgml^−1^) showed no activity, whereas, both microspheres without *β*CD and microspheres containing *β*CD led to a marked and significant (*P* < 0.01) decline in *P. dicentrarchi* survival, at both microsphere concentrations tested (10 and 50 µg ml^−1^). The decline obtained with *β*CD-containing microspheres was significantly greater than other treatments. They proposed that glutaraldehyde, which used as a cross linking agent, had a strong antiparasitic activity. More recently, relative impacts of chitosan, alum, and FeCl_3_ coagulation on the removal of *Cryptosporidium parvum* oocysts were investigated previously (10). At all doses investigated, chitosan coagulation resulted in excellent turbidity and particle reductions by filtration. Nonetheless, chitosan coagulation at doses of 0.1, 0.5, and 1.0 mg L^−1^ did not result in appreciable improvements in *C. parvum* oocyst removal and complete coagulation failure.

The functional properties of chitosan are likely to differ based on the preparation methods used to convert chitin to chitosan and influenced by intrinsic and extrinsic factors such as the type of chitosan (e.g. plain or derivative), molecular weight, degree of deacetylation, and environmental conditions (6). For example, it has been proposed that low molecular weight chitosans (of less than 10 kDa) have greater antimicrobial activity (15). Therefore, there is a need to study the efficiency of different types of chitosan derivatives on *T. gallinae* *in vitro* and in bird model alone and/or with combination with other agents.
